# Clays as Dual-Function Materials for TNT Adsorption and Catalytic Degradation: An Experimental Approach

**DOI:** 10.3390/ma18163824

**Published:** 2025-08-14

**Authors:** Raluca Florenta Doroftei, Diana Mirila, Mihaela Silion, Daniela Ionita, Ana-Maria Rosu, Corneliu Munteanu, Bogdan Istrate, Gabriela Muntianu, Ana-Maria Georgescu, Ileana-Denisa Nistor

**Affiliations:** 1 School of Doctoral Studies, “Vasile Alecsandri” University of Bacău, 157 Calea Marasesti Street, 600115 Bacău, Romania; ralucadoroftei@yahoo.com; 2 Faculty of Engineering, Department of Environmental Engineering, Mechanical Engineering and Agritourism, “Vasile Alecsandri” University of Bacău, 157 Calea Marasesti Street, 600115 Bacău, Romania; 3 Physics of Polymers and Polymeric Materials, “Petru Poni” Institute of Macromolecular Chemistry, 41A Grigore Ghica Voda Alley, 700487 Iasi, Romania; silion.mihaela@icmpp.ro (M.S.); ionita.daniela@icmpp.ro (D.I.); 4 Faculty of Engineering, Department of Chemical and Food Engineering, “Vasile Alecsandri” University of Bacău, 157 Calea Marasesti Street, 600115 Bacău, Romania; ana.rosu@ub.ro (A.-M.R.); muntianu.gabriela@ub.ro (G.M.); 5 Faculty of Mechanical Engineering, Department of Mechanical, Mechatronics and Robotics Engineering, “Gheorghe Asachi” Technical University of Iasi, Dimitrie Mangeron Boulevard, No. 43, 700050 Iasi, Romania; corneliu.munteanu@academic.tuiasi.ro (C.M.); bogdan.istrate@academic.tuiasi.ro (B.I.)

**Keywords:** adsorption, catalytic destruction, 2,4,6-trinitrotoluene, pillared clays, vanadium

## Abstract

This study explores the adsorption and catalytic degradation of 2,4,6-trinitrotoluene (TNT) from aqueous solutions, using montmorillonite-based catalysts. Commercially, montmorillonite K10 was modified through aluminum pillaring (K10-Al-PILC), followed by vanadium intercalation (K10-Al-PILC-V) and ozone activation. A novel aspect of this work is the use of naturally contaminated water as the TNT source. The selected sample, collected from the Plaiul Arșiței–Cireșu–Leșunț region (Oituz, Bacau, Romania), originated from an area historically exposed to explosive residues, where TNT traces were previously identified. The adsorption performance of the materials was evaluated by varying adsorbent dosage, contact time, and solution pH. Catalytic ozonation experiments were conducted under different catalyst masses, ozone concentrations, and reaction times to assess degradation efficiency. The results demonstrated that aluminum pillaring significantly enhanced the adsorption capacity of the clay, while vanadium incorporation further improved both adsorption and catalytic activity. The vanadium-modified material exhibited superior performance in TNT removal, both through adsorption and oxidative degradation. Additionally, the catalytic ozonation process led to the formation of degradation products with reduced toxicity, confirming the potential of these materials for environmental remediation of nitroaromatic pollutants in real water systems.

## 1. Introduction

**Figure 1:**
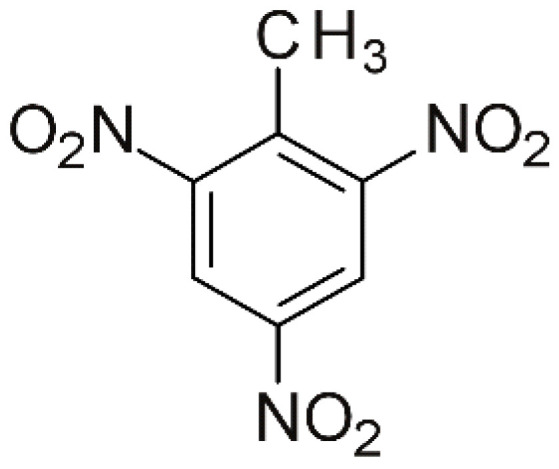
The molecular formula of 2,4,6-trinitrotoluene.

2,4,6-trinitrotoluene (TNT), presented in Figure 1, is a nitroaromatic compound predominantly used as a high-energy material, particularly in military applications and industrial applications. TNT-based waste, dispersed because of armed conflicts or military exercises, contains toxic substances such as heavy metals (lead, mercury, cadmium), unreacted energetic materials, and hazardous secondary chemical compounds [1]. These substances infiltrate the soil and leach into groundwater, thereby posing a serious threat to human health and biodiversity.

Explosive war remnants remain a significant threat, particularly in former battlefields now embedded in villages, farmland, and along popular recreation trails. Because explosive ordnance disposal (EOD) teams can recover munitions only when access and safety conditions allow, many items are destroyed in situ, leaving nitroaromatic residues that leach into local soils and waterways (as documented in recent media reports [2,3]). These areas are frequented by residents and tourists alike, making the issue both a public health and an ecological concern.

Additionally, by-products such as heavy metals and fine particles contribute to air pollution and the formation of acid rain. Despite their harmful effects [4], nitroaromatic compounds continue to be widely used in the production of pharmaceuticals, pigments, polymers, and pesticides, with an annual output exceeding one hundred million tons [5,6]. TNT is considered a cost-effective option in industrial and chemical engineering sectors, where direct exposure of workers (through inhalation and dermal contact) as well as environmental exposure (through waste discharge) is virtually unavoidable. Furthermore, contamination of surface and groundwater with TNT and 2-ADNT (2-amino-4,6-dinitrotoluene) has been reported [7], originating from polluted soils, wastewater from military industry sites, and freshwater lakes, because of munitions dumping and related waste. Consequently, the widespread presence of TNT in the environment significantly increases the risk of both short-term and long-term toxic effects on human health and wildlife [8,9].

Thus, the removal of TNT from the water system has become mandatory due to concerns related to public security, civilian safety, and environmental protection. A variety of treatment methods have been proposed and developed, including oxidation [10], reduction, photo transformation [11], and adsorption [12,13]. Among these, adsorption is considered the most promising approach due to its operational simplicity and minimal generation of secondary pollutants. To address these challenges, the use of unconventional materials such as montmorillonite clays has emerged as a promising solution. Owing to their high adsorption capacity [14,15], these clays can retain nitroaromatic pollutants, toxic organic compounds, and other hazardous substances from water, thus preventing the contamination of rivers and aquifers. Unlike conventional filtration methods, which are often associated with high costs and advanced technological requirements, the use of clays is economically accessible, renewable, and environmentally sustainable [16,17]. Montmorillonite, due to its layered structure and large specific surface area, can adsorb pollutant molecules through ion exchange and electrostatic interactions. These characteristics make clays effective alternatives for the retention of such pollutants. The adsorption yield (*η_ads_*) is a key performance indicator for evaluating the capacity of an adsorbent to remove a target contaminant from aqueous media. It is calculated using the following equation:

(1) $$\eta_{ads}=\frac{A_{t_{0}}-A_{t_{1}}}{A_{t_{0}}}\times 100$$

where $A_{t_{0}}$ is the initial absorbance of the contaminant at moment *t*_0_, $A_{t_{1}}$ is the final absorbance after the adsorption process at moment *t*_1_, and *η_ads_* represents the adsorption yield (%).

A higher efficiency value indicates more effective adsorption behavior. This parameter is widely used in environmental studies [18,19,20] to quantify pollutant removal under various experimental conditions.

Catalytic ozonation represents an advanced oxidation process (AOP) that utilizes ozone in conjunction with a solid catalyst to produce hydroxyl radicals (•OH), which are highly reactive and non-selective oxidizing agents. This method has demonstrated significant efficiency in breaking down persistent organic contaminants, such as 2,4,6-trinitrotoluene (TNT), which are typically resistant to conventional treatment technologies [10,21,22]. Vanadium-based catalysts have proven effective in oxidation processes applied to environmental pollutants, including selective oxidation reactions involving alkanes, aromatic compounds, and alcohols [12,23,24]. The risk of TNT contamination is higher when there is groundwater nearby [25]. There is also a risk in sandy soil that does not contain organic material, as TNT will not degrade as quickly and can migrate into groundwater or leach into surface waters [26,27].

This study investigates the adsorption performance of montmorillonite-derived clay materials, specifically aluminum-pillared montmorillonite (K10-Al-PILC) and its vanadium-intercalated counterpart (K10-Al-PILC-V), for the removal of 2,4,6-trinitrotoluene (TNT) from aqueous media. The materials are further evaluated in a catalytic ozonation [28] process using K10-Al-PILC-V to enhance TNT degradation through advanced oxidation.

The scientific goal of this study is to develop and evaluate structurally modified montmorillonite-based materials that combine adsorption and catalytic activity for efficient TNT removal. By integrating aluminum pillaring and vanadium incorporation, the materials exhibit dual functionality, enhancing both adsorption capacity and catalytic ozonation efficiency. This approach offers innovative and sustainable solutions for water purification, with potential applications in the treatment of wastewater from explosives manufacturing, military facilities, and related industries.

## 2. Materials and Methods

All chemicals used in this study, including sodium hydroxide (NaOH, 39.997 g·mol^−1^), aluminum chloride (AlCl_3_, 133.34 g·mol^−1^), silver nitrate (AgNO_3_, 169.87 g·mol^−1^), K10 montmorillonite, and vanadyl sulfate (O_5_SV, 163 g·mol^−1^), were of analytical grade and purchased from Sigma-Aldrich. Throughout the experimental procedures, double-distilled water was used. The TNT analyzed in this work originated from contaminated water samples collected from various locations in Bacău County, Romania (Table 1), where traces of 2,4,6-trinitrotoluene were detected near deteriorated munitions.

**Figure 2:**
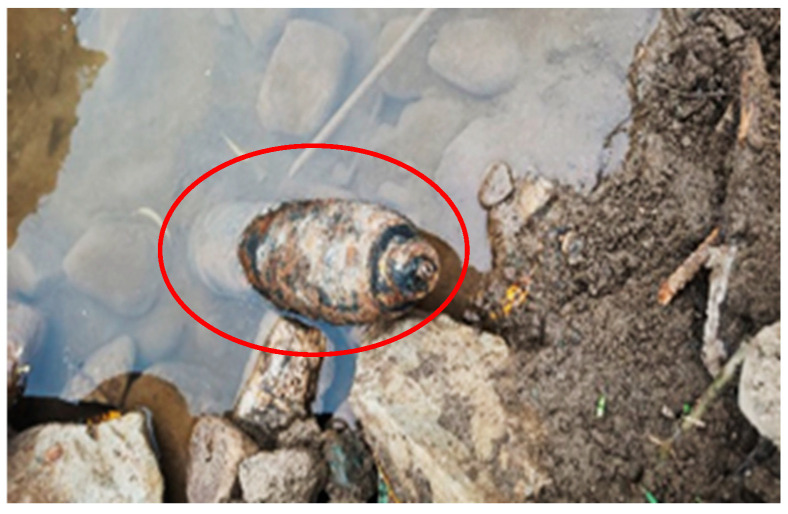
Source of TNT from abandoned unexploded ordnance (UXO) discovered in Plaiul Arșiței-Cireșu-Leșunț, Oituz, April 2025.

Traces of TNT were identified in several water sources; however, only one sample was selected for detailed analysis. The chosen sample, collected in April 2025 from source S5 (Figure 2) in the Plaiul Arșiței–Cireșu–Leșunț region (Oituz), corresponds to an area considered at increased risk of TNT contamination. This risk is presumed to arise from the prolonged degradation of residual explosive compounds [1], historically present in the vicinity. Such compounds can persist in the environment and gradually leach into the surface or groundwater, contributing to long-term pollution. The presence of TNT in aquatic systems not only poses toxicological risks to living organisms but also compromises water quality and threatens the ecological balance of affected areas.

The identification of TNT and its transformation metabolites in the water sample S5 was performed using HPLC-MS. Since TNT is not commercially accessible for research purposes in Romania, compound identification was achieved through comparison of the obtained mass spectrometric data with values reported in the scientific literature [29,30,31].

### 2.1. Pretreatment of Water Samples

To potentially inhibit microbial activity in water samples, two distinct pretreatment protocols were implemented, each designed to suppress the metabolic activity of TNT-degrading bacterial strains while preserving the chemical integrity of the target molecule.

Several bacterial strains have been reported in the literature for their ability to degrade TNT, including *Klebsiella variicola* [32], *Stenotrophomonas* sp. SG1 (a Gram-negative bacterium) [33], and *Pseudomonas* sp. [34], as well as other efficient degraders, such as *Clostridium* spp. [35]. These microorganisms utilize various enzymatic pathways to transform TNT into less toxic intermediates, contributing to its bioremediation in contaminated environments [36,37]. 

Sample S5 was subjected to thermal treatment at 70 °C, as indicated in Table 1. This step was necessary due to the stagnant nature of the source and its high microbiological potential, ensuring the inactivation of mesophilic bacteria while preserving TNT integrity. Water temperature is a key regulator of microbial metabolism involved in the biodegradation of organic compounds, including TNT. In cold mountain streams (7–12 °C) microbial activity is markedly reduced, whereas in stagnant, swamp-like waters, summer temperatures may exceed 25 °C, promoting intense bacterial activity and accelerated degradation of sensitive molecules such as TNT in the absence of adequate preservation [38]. For samples taken from cold, fast-flowing sources, freezing at −20 °C for 48 h was considered sufficient to inhibit biological activity, given the low risk of rapid biodegradation and the structural stability of TNT at low temperature. For stagnant sources with high microbiological potential, a thermal treatment at 70 °C for 30 min was applied, effectively inactivating mesophilic bacteria while preserving TNT integrity for subsequent analysis.

In well-oxygenated, high-energy waters such as rivers and brooks, microorganism counts are relatively low [39] and TNT is not rapidly degraded by abiotic reactions. In stagnant waters, degradation may be accelerated by anaerobic bacteria, but this can increase the risk of bioaccumulation. TNT is only sparingly soluble in water (≈100–200 mg L^−1^ at 20 °C [40]), favoring precipitation or sorption onto suspended particles; a significant fraction therefore reaches underlying sediments or soils where further degradation can occur [41]. When soil is chronically exposed to an external TNT source (e.g., decaying ordnance), biodegradation occurs but is often outpaced by the input flux, leading to pollutant accumulation. Although adapted microbiota may develop, they rarely achieve complete removal without human intervention. Over time, TNT can migrate to the groundwater table or bind to humic or clay particles, decreasing its bioavailability but not its long-term toxicity [42].

### 2.2. Synthesis of Materials

The synthesis of the chemically modified clay material (K10-Al-clay) was initiated by subjecting commercial montmorillonite K10. The clay was modified through ex situ pillaring with Keggin-type polyoxocations, following the method described by Cao et al. [43]. A molar ratio of OH^−^ to Al^3+^ of 2.4 was used to prepare the pillaring solution, employing two precursor solutions: 0.4 M NaOH (designated as Solution A) and 0.4 M AlCl_3_·6H_2_O (Solution B). Solution A was added dropwise to Solution B at a controlled rate of 0.5 mL·min^−1^ under continuous stirring at 40 °C. The resulting mixture was then subjected to an ageing process for 18 h at 65 °C. The resulting polyoxocation solution was intercalated into the K10 montmorillonite via gradual addition with stirring for 2 h at 40 °C, followed by 3 h of homogenization. To obtain the pillaring solution, the mixture was subjected to a microwave irradiation aging method, according to [44], at 160 W for 15 min. The final material was washed, centrifuged, and dried sequentially at 40 °C, 60 °C, 90 °C, and 120 °C. Calcination was performed at 430 °C for 2 h to obtain the K10-Al-PILC.

Two grams of K10-Al-PILC powder was added to 150 mL of 3 mmol VOSO_4_·H_2_O solution. This suspension was mixed under continuous magnetic stirring for 24 h at 50 °C. The mixture was then centrifuged and washed with deionized water to remove residual SO_4_^2−^ ions, confirmed by testing the supernatant with 0.2 N BaCl_2_. The impregnated material was dried for 3 h at 30 °C and denoted as K10-Al-PILC-V. The procedure was established in accordance with the relevant literature [24] on PILC synthesis and vanadium impregnation methods.

### 2.3. Devices

BET surface area and nitrogen adsorption–desorption isotherms were recorded at 77.35 K over a relative pressure range of P/P_0_ = 0.005–1.0 using a NOVA 2200e Gas Sorption Analyzer manufactured by Quantachrome Instruments (Boynton Beach, FL, USA). Data processing was performed with the NovaWin software, version 11.03. Prior to analysis, the samples were degassed under vacuum at 160 °C for 4 h to eliminate physisorbed contaminants. The specific surface area and total pore volume were calculated from the volume of nitrogen desorbed at a relative pressure close to saturation (P/P_0_ ≈ 1), using the Barrett–Joyner–Halenda (BJH) method applied to the desorption branch of the isotherm. This branch was also used to determine the pore size distribution and mesopore volume, based on the BJH method.

The X-ray diffraction patterns of the samples were measured using a SmartLab X-ray diffractometer manufactured by Rigaku Corporation (Akishima-shi, Tokyo, Japan), in Bragg–Bretano geometry, with a Cu anode (X-ray wavelength of 1.5406 Å), over an angular range of 2–40° (2θ), using a scanning step of 0.02° and a recording rate of 3°/min.

FTIR analysis was conducted using an IRSpirit spectrometer manufactured by Shimadzu Corporation (Kyoto, Japan), equipped with a single-accessory attenuated total reflectance (ATR) module. The measurements were performed in transmittance mode, with a scan rate of 45 scans per minute, a spectral resolution of 8 cm^−1^, and a scanning range covering 400 to 5000 cm^−1^.

The surface morphology and elemental composition of the specimens were examined using a Verios G4 UC scanning electron microscope (Thermo Fisher Scientific, Waltham, MS, USA) equipped with an energy-dispersive X-ray spectroscopy (EDS) system featuring an Octane Elect Super SDD detector (Mahwah, NJ, USA). To enhance electrical conductivity and minimize charging effects during electron beam exposure, a 6 nm platinum coating was applied using a Leica EM ACE200 sputter coater (Leica Microsystems, Wetzlar, Germany). SEM imaging was conducted with a backscattered electron detector (Mirror Detector—MD) at an accelerating voltage of 15 kV.

Thermogravimetric analysis (TGA) was carried out using a TGA 5500 system (TA Instruments, New Castle, DE, USA). Each sample, weighing approximately 6.45 mg, was placed in a platinum crucible and subjected to a controlled heating program under a nitrogen flow (25 mL·min^−1^), with a temperature ramp of 10 °C·min^−1^, reaching a final temperature between 600 and 700 °C. Throughout the process, mass loss was continuously monitored. High-performance liquid chromatography coupled with mass spectrometry (HPLC-MS) was performed using an Agilent 6500 Series Accurate-Mass Quadrupole Time-of-Flight (Q-TOF) LC/MS system (Agilent Technologies, Santa Clara, CA, USA). For the analysis of the water sample (S5), an Agilent 1200 Series HPLC system was employed, equipped with a reversed-phase Prontosil C18 column (125 mm × 4.6 mm, 5 μm particle size). Chromatographic separation was optimized under the following conditions: the column temperature was maintained at 30 °C, the injection volume was set to 50 µL, and the flow rate was adjusted to 1 mL·min^−1^, with a 9:1 split directed to the electrospray ionization mass spectrometer (ESI-MS). A gradient elution program was used to achieve compound separation.

The mobile phase initially consisted of 80% solvent A (water) and 20% solvent B (acetonitrile). After 10 min, the proportion of solvent B was gradually increased to 100%, followed by a return to the initial 20% within the next 5 min. The total run time was 15 min, after which the column was re-equilibrated for 5 min before subsequent injections. All solvents were pre-filtered and degassed prior to use. Detection of TNT and its transformation products was performed using both a diode array detector (UV-VIS DAD, Berlin, Germany) set at 230 nm and a mass spectrometer (Agilent Technologies, Santa Clara, CA, USA). For compound identification, electrospray ionization (ESI) in negative mode was employed. The Q-TOF MS parameters were as follows: nitrogen drying gas at 8 L·min^−1^, drying temperature of 325 °C, nebulizer pressure of 35 psig, capillary voltage set to 4000 V, and fragmentor voltage at 175 V. The mass range scanned was m/z 80–1500. Data acquisition and analysis were carried out using MassHunter Workstation Software, version B.07.00 (Agilent Technologies, USA).

## 3. Results

### 3.1. Materials Analysis

The commercial material K10 and the synthesized adsorbents K10-Al-PILC and K10-Al-PILC-V were characterized using a range of analytical techniques to assess their structural, textural, and morphological properties.

#### 3.1.1. Brunauer–Emmett–Teller (BET) Analysis

Brunauer–Emmett–Teller (BET) analysis was employed to determine the specific surface area, total pore volume, and average pore diameter of the synthesized materials, with the results presented in Table 2. The specific surface area measured for the raw clay was 242.11 m^2^·g^−1^, which closely aligns with the value reported by the manufacturer in the technical documentation [45].

Following the pillaring process, a decrease in the surface area of the synthesized material (K10-Al-PILC) was observed, consistent with findings reported in previous studies [44,46,47]. This reduction may be attributed to coke deposition, which could block the pores or obstruct pore entrances during calcination, or to incomplete decomposition of precursor compounds. Additionally, the incorporation of vanadium led to a further decline in BET surface area, from approximately 186 m^2^·g^−1^ to 177 m^2^·g^−1^, suggesting partial coverage or blockage of surface-active sites. This decrease implies potential pore blockage by vanadium species and/or the formation of larger space-occupying structures that restrict nitrogen access to previously available micropores. Additionally, the total pore volume decreased from 0.171 to 0.158 cm^3^·g^−1^, further supporting the hypothesis of partial pore filling or local structural densification induced by vanadium. A slight increase in average pore diameter, from 4.549 nm to 4.564 nm, was also observed. This shift is significant at the nanoscale and can be attributed to a reduced presence of smaller pores (blocked or narrowed by vanadium), thereby shifting the distribution toward larger mesopores or facilitating the formation of broader pore structures.

**Figure 3:**
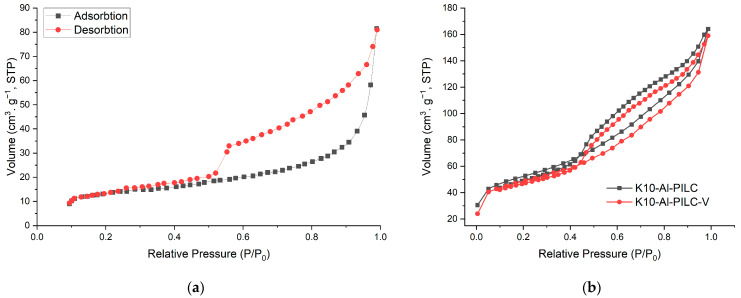
N_2_ adsorption-desorption isotherm plots for K10 [48]: (**a**) K10-Al-PILC and (**b**) K10-Al-PILC-V.

**Figure 4:**
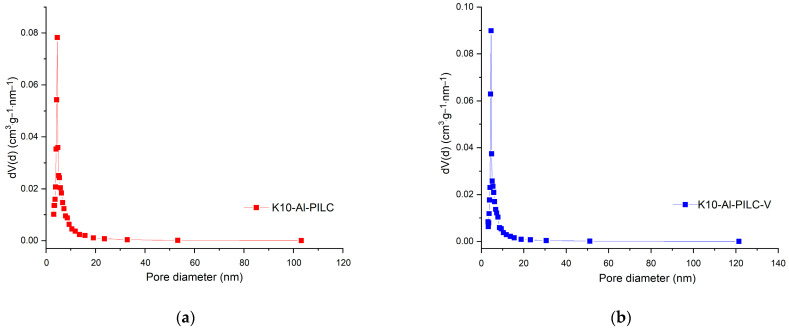
Pore size distribution curves for (**a**) K10-Al-PILC and (**b**) K10-Al-PILC-V.

K10-Al-PILC and K10-Al-PILC-V exhibit type IV isotherms with H3-type hysteresis loops (Figure 3a), confirming their mesoporous nature and slit-shaped pore morphology, characteristic of pillared clays with layered structures. Both materials exhibit adsorption behavior typical of mesoporous structures. While K10-Al-PILC demonstrates slightly higher adsorption capacity, K10-Al-PILC-V shows a comparable overall trend. Despite a modest reduction in performance, the structural features of K10-Al-PILC-V remain favorable, potentially offering improved accessibility for larger molecules. The incorporation of vanadium leads to a slight reduction in BET surface area, approximately 7.6%, yet the overall mesoporous structure of K10-Al-PILC-V remains largely intact. The moderate increase in average pore diameter improves the material’s accessibility to larger organic molecules, such as TNT, which can benefit diffusion and retention within the porous network. Importantly, the introduction of vanadium contributes additional Lewis acidic and redox-active sites, enhancing both the adsorption capacity and the material’s catalytic behavior, particularly in oxidative environments. This is reflected in the superior performance of K10-Al-PILC-V under combined adsorption-ozonation conditions, where it achieves higher removal efficiency compared to the vanadium-free counterpart. These findings suggest that adsorption efficiency is influenced not only by surface area but also by the chemical nature and distribution of active sites, which play a critical role in pollutant interaction and transformation. The pore size distribution curves for K10-Al-PILC and K10-Al-PILC-V, shown in Figure 4, were obtained from the desorption branch of the nitrogen adsorption–desorption isotherms using the Barrett–Joyner–Halenda (BJH) method. Both materials display mesoporous structures, with pore diameters primarily falling within the 2–50 nm range, in agreement with the findings illustrated in Figure 3.

#### 3.1.2. X-Ray Diffraction (XRD) Analysis

**Figure 5:**
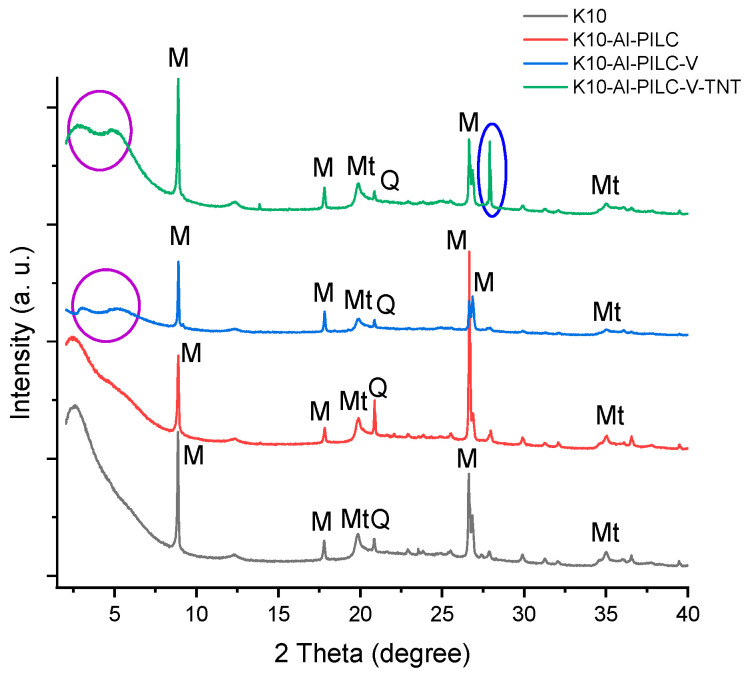
XRD analysis of materials (Mt—montmorillonite, M—muscovite, Q—quartz).

XRD analysis was employed to investigate the structural changes in montmorillonite-based materials after aluminum pillaring and vanadium intercalation. The diffractogram of the K10 material (Figure 5) revealed the presence of montmorillonite (Mt) as the main crystalline phase, along with muscovite (M) and quartz (Q) as natural impurities. These phases are typical of clays and confirm the layered structure characteristic of montmorillonite.

For the K10-Al-PILC material, no new diffraction peaks were observed; however, changes in the intensity of existing peaks were recorded. These variations suggest a structural rearrangement of the clay layers without the formation of new detectable crystalline phases. This behavior is typical of pillared materials, where the intercalation of aluminum polyoxocations stabilizes the layered architecture without generating distinct crystalline compounds.

The X-ray diffraction pattern of the K10-Al-PILC-V material revealed two additional reflections at approximately 2.8° and 4.1° (2θ), which are indicative of vanadium species incorporated within the pillared clay framework. These peaks indicate the formation of poorly defined crystalline phases or intercalated structures, rather than well-defined vanadium oxides. Thus, vanadium appears to be dispersed within the clay matrix, contributing to modifications in the interlayer structure. The K10-Al-PILC-V material was regenerated two times after its contact with TNT; the third time the adsorption and degradation efficiency dropped below 50%. That is why the regeneration was not continued. After contact with TNT, the K10-Al-PILC-V-TNT sample showed an additional peak at 2θ ≈ 27.9°, initially attributed to adsorbed TNT or its transformation products. However, this peak also appears, though with lower intensity, in the other samples, suggesting that the signal may be influenced by incomplete grinding or a common structural feature. Moreover, the very low TNT concentration may have limited the visibility of its crystalline signature in the XRD pattern. Moreover, the intensification of the peaks at approximately 3° and 5° suggests a significant structural interaction between TNT and the catalytic material, possibly through complex formation in the intercalated network.

Overall, the XRD analysis confirms the structural stability of montmorillonite following chemical modifications and highlights the effects of vanadium intercalation and TNT exposure on the crystalline structure. These findings support the effectiveness of the synthesized materials in TNT adsorption and catalytic degradation processes.

#### 3.1.3. Fourier Transform Infrared Spectroscopy (FTIR) Analysis

**Figure 6:**
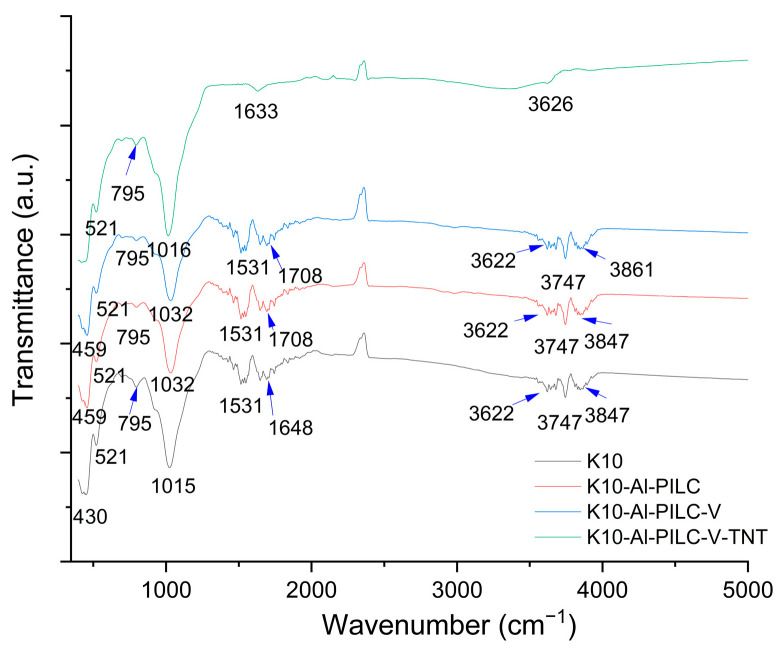
FTIR analysis of materials.

The FTIR spectra (Figure 6) of the four analyzed materials, K10, K10-Al-PILC, K10-Al-PILC-V and K10-Al-PILC-V-TNT, reveal the progressive structural modifications induced by aluminum pillaring and subsequent vanadium incorporation. For the raw K10 clay, a broad absorption band around 3400 cm^−1^ is observed, corresponding to the O–H stretching vibrations of interlayer water and structural hydroxyl groups. The band near 1630 cm^−1^ is attributed to the H–O–H bending mode of absorbed water. A strong band in the 1000–1100 cm^−1^ region is characteristic of Si–O stretching vibrations in the tetrahedral silicate layers, while bands below 600 cm^−1^ are associated with Si–O–Al and Si–O–Si bending modes, typical of the montmorillonite framework [49]. In the case of K10-Al-PILC, the FTIR spectrum exhibits significant changes. The Si–O stretching band becomes broader and slightly shifts, indicating structural distortion due to the intercalation of Keggin-type aluminum polyoxocations. Additional bands or shoulders in the 600–800 cm^−1^ region suggest the formation of Al–O–Al and Al–O–Si linkages, confirming the successful pillaring process [50] and the formation of a stable inorganic framework [51]. For the K10-Al-PILC-V sample, the spectrum retains the main features of the pillared structure, indicating that the aluminum framework remains intact after vanadium incorporation. Subtle shifts in the 900–1000 cm^−1^ region may be attributed to V=O stretching vibrations or V–O–Al interactions, suggesting the presence of vanadium species anchored to the clay surface or interlayer. These modifications are consistent with the introduction of redox-active sites, which enhance the material’s catalytic potential [52,53].

According to the FTIR analysis of the material recovered after TNT adsorption, no characteristic peaks of this pollutant were identified. In the literature, TNT-specific peaks are observable when the compound is in its standard form [54]. However, in the present study, the TNT source was not standard, as confirmed by HPLC analysis, which revealed the presence of additional metabolites. Therefore, TNT could not be clearly detected, likely due to its concentration being below the minimum detection limit of the device used. Overall, the FTIR analysis confirms the stepwise structural evolution from raw montmorillonite to aluminum-pillared and vanadium-functionalized clays. These transformations are essential for improving the materials’ adsorption capacity and catalytic performance.

#### 3.1.4. Scanning Electron Microscopy (SEM) Analysis

**Figure 7:**
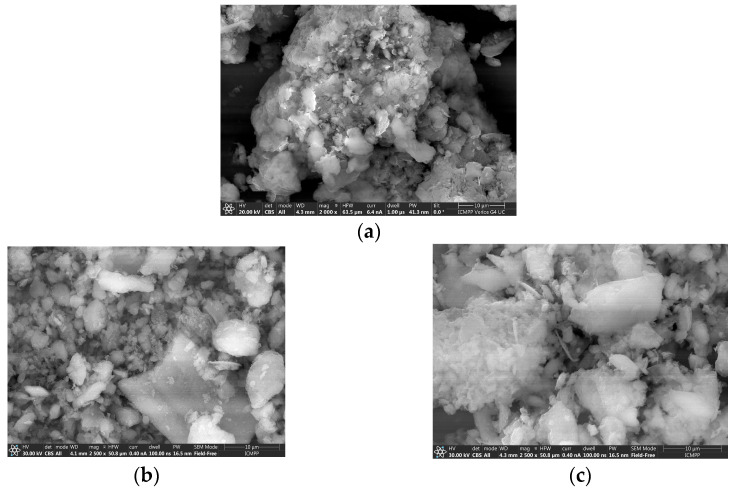
SEM analysis of (**a**) K10; (**b**) K10-Al-PILC; (**c**) K10-Al-PILC-V.

SEM provides essential insights into the surface morphology and microstructural characteristics of the synthesized materials, enabling a detailed comparison of textural features, particle distribution, and structural organization resulting from different synthesis pathways. The SEM analysis of K10-Al-PILC presented in Figure 7b reveals a heterogeneous surface morphology characterized by irregularly shaped particles with varying sizes and a moderately rough texture.

The particles exhibit partial agglomeration and a complex surface structure, indicating a porous material with microstructural development. These features are typical of pillared clays derived from commercial K10 montmorillonite (Figure 7a), where the initial structural modifications influence the aluminum pillaring process, resulting in a less uniform distribution of pillars and surface features. In contrast, the SEM image of K10-Al-PILC-V (Figure 7c) shows a more homogeneous morphology, with well-defined particles that are more uniformly dispersed and exhibit reduced agglomeration. The surface appears smoother and more compact, suggesting improved structural organization. Vanadium, introduced through impregnation, appears to enhance particle dispersion and stabilization, reducing surface charge accumulation and contributing to an optimal structural organization of the material.

#### 3.1.5. Energy Dispersive X-Ray (EDX) Spectroscopy

**Figure 8:**
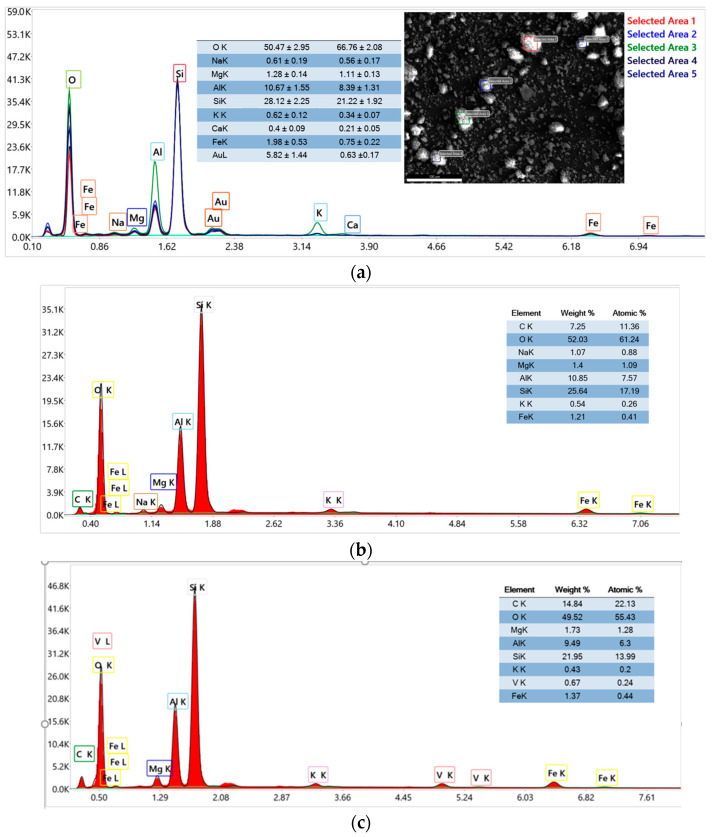
EDX analysis of (**a**) K10; (**b**) K10-Al-PILC; (**c**) K10-Al-PILC-V.

The EDX analysis of raw material K10 was performed at five different areas, as shown in Figure 8a. The EDX analysis of the K10-Al-PILC sample (Figure 8) confirms the successful incorporation of aluminum into the clay structure, as evidenced by the elevated aluminum content relative to silicon. This increase in the Al/Si ratio (from 0.24 in the case of raw material to 0.42 for K10-Al-PILC) is indicative of the formation of Al-based pillars within the interlayer space of the montmorillonite framework. The elemental profile is dominated by oxygen and silicon, consistent with the layered silicate structure of the clay, while minor elements such as magnesium and potassium reflect the natural composition of the raw material.

In contrast, the EDX spectrum of the K10-Al-PILC-V sample reveals the presence of vanadium, detected at approximately 0.67 wt%, confirming its successful impregnation onto the pillared clay. The aluminum content remains significant (9.49 wt%), indicating that the pillared structure is preserved following vanadium modification.

The presence of vanadium, alongside aluminum, introduces redox-active sites that are expected to enhance the material’s catalytic performance, particularly in oxidative processes such as ozonation. The presence of aluminum (Al) and vanadium (V) in the materials K10-Al-PILC, K10-Al-PILC-V, and K10-Al-PILC-V-TNT is confirmed by the semi-quantitative data presented in the tables included in Figure 8a–c. Aluminum plays a key role in stabilizing the pillared structure and enhancing the surface area and acidity of the material, which are critical for catalytic activity. Vanadium, on the other hand, acts as an active redox center, facilitating the generation of reactive oxygen species (ROS) during ozonation and catalytic ozonation processes, thereby improving the degradation efficiency of organic pollutants.

Overall, the comparative EDX data support the structural evolution from a purely aluminum-pillared clay to a bifunctional material incorporating both aluminum and vanadium. This transformation is essential for enabling dual functionality: adsorption and catalysis, in environmental remediation applications.

#### 3.1.6. Thermogravimetric Analysis (TGA)

**Figure 9:**
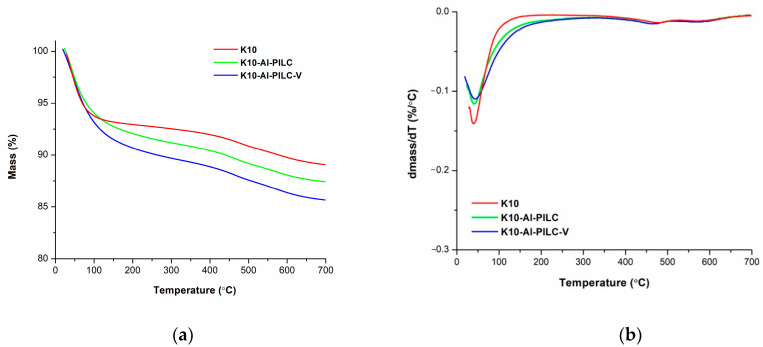
Thermogravimetric Analysis (TGA) (**a**) and Derivative Thermogravimetric (DTG) (**b**) curves of K10, K10-Al-PILC, and K10-Al-PILC-V samples.

TGA and DTG analysis were employed to evaluate the thermal stability of the synthesized materials. The corresponding DTG curves were derived by operating a first-order derivative of the experimental TGA data (Figure 9).

The thermal decomposition of the analyzed materials occurred in three distinct stages. For each stage, the onset and completion temperatures (T_1_ and T_2_), the corresponding weight loss (Δm_i_), and the temperature at which the maximum rate of thermal degradation occurred (T_max_) were determined. These values are summarized in Table 3.

The thermal decomposition profile of the three samples follows a similar pattern, consisting of three distinct stages. The most significant mass loss, approximately 10%, occurs during the first stage, primarily due to water release. In contrast, the second and third stages are characterized by weight losses of less than 2%. However, a notable difference in the first-stage mass loss is observed for the clay materials derived from montmorillonite. Specifically, K10-Al-PILC and K10-Al-PILC-V exhibit a broader temperature range in the initial decomposition stage compared to the unmodified K10 sample. This effect may be due to the chemical modification of the clay. Thus, aluminum pillaring and vanadium incorporation do not significantly modify the thermal behavior compared to K10, but they do affect the intensity of water removal from the synthesized adsorbents.

Thermal stability is a critical parameter for materials intended for use under variable temperature conditions, while adsorption capacity reflects the material’s effectiveness in capturing and degrading contaminants. The stable thermal response observed indicates that the materials can be applied in industrial processes without undergoing significant degradation, an essential factor for ensuring long-term durability and operational efficiency.

#### 3.1.7. High-Performance Liquid Chromatography with Ultraviolet Detection (HPLC–UV) and Electrospray Ionization Mass Spectrometry (ESI-MS)

**Figure 10:**
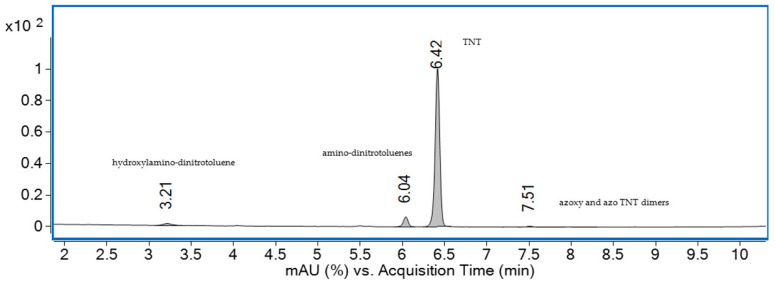
HPLC–UV chromatogram at 230 nm for the water sample collected from S5 source.

The chromatographic separation of TNT and its metabolites from water sample S5 is presented in Figure 10.

**Figure 11:**
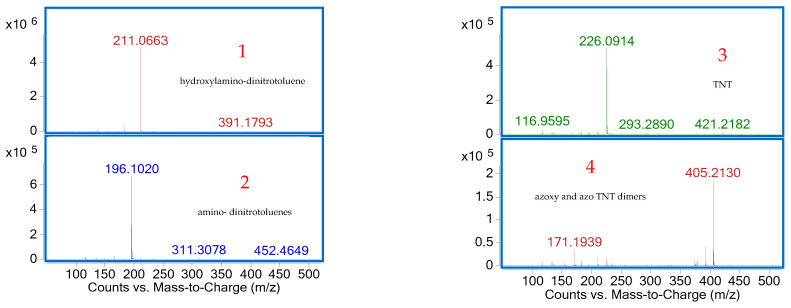
Negative ESI-MS spectra of compounds 1-4 identified in water sample S5 after HPLC separation.

Four peaks were observed in the HPLC–UV chromatogram, and negative ESI-MS analysis of each peak enabled the detection of TNT and three of its metabolites, based on the identification of their deprotonated molecular ions [M–H]^−^ (Figure 11).

The first peak, peak 1, which eluted at 3.21 min with an *m*/*z* value of 211, was identified as hydroxylamino-dinitrotoluene, while peak 2, detected at 6.04 min with an *m*/*z* of 196, was attributed to amino-dinitrotoluenes, suggesting nitro group reduction [55]. Peak 3, with a retention time of 6.42 min and an *m*/*z* of 226, corresponded to TNT, confirming its presence in water sample S5. Finally, peak 4, observed at 7.51 min with an *m*/*z* of 405, was assigned to azoxy and azo TNT dimers, compounds typically formed through reduction reactions followed by photochemical transformation [29].

These results confirm the presence of TNT in the water sample, along with the formation of three metabolites associated with biological processes occurring in contaminated areas [56].

Although the exact speciation of TNT in the water samples was not directly measured, the historical context of the contamination provides important insight. Given that the unexploded ordnance (UXO) in the Plaiul Arșiței–Cireșu–Leșunț area dates back to the First World War, the explosive charge was most likely composed of either nearly pure TNT (Sprengstoff 12) or an Amatol blend, typically 60/40 or 80/20 ammonium nitrate/TNT. Over time, the highly soluble ammonium nitrate fraction leaches out, leaving behind unmetallized TNT as the dominant residual compound [57,58].

Under the specific physico-chemical conditions of source S5, this residue dissolves primarily as neutral TNT and is partially reduced in situ to 2-ADNT and 4-ADNT, forming dissolved, bioavailable species rather than stable crystalline or colloidal phases [59,60]. These transformation products are of particular concern due to their persistence and potential toxicity.

To ensure accurate quantification of these species, our field protocol focused on preserving the total dissolved fraction of TNT and ADNT through immediate thermal stabilization and subsequent analysis. This approach allowed us to capture the environmentally relevant forms of contamination and better assess the efficiency of the proposed remediation strategies.

### 3.2. TNT Adsorption

The adsorption performance of the synthesized clay-based materials was systematically investigated under various experimental conditions, including adsorbent type, contact time, and solution pH, in order to evaluate their efficiency in removing TNT from contaminated aqueous media. It is important to note that the vanadium-functionalized material (K10-Al-PILC-V) was not evaluated in the adsorption experiments, as vanadium incorporation primarily enhances the material’s catalytic performance during ozonation, rather than its direct adsorption capacity.

#### 3.2.1. The Influence of Adsorbent Mass

**Figure 12:**
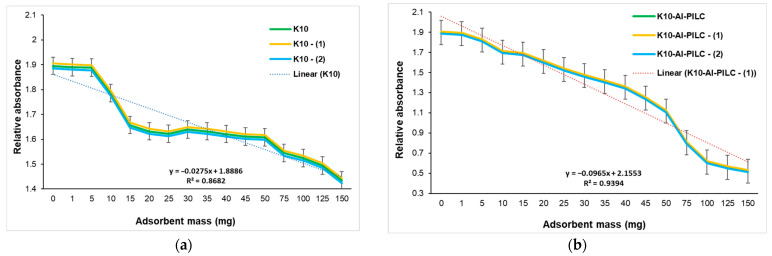
Influence of adsorbent mass on TNT absorption at 232 nm from S5 sample onto (**a**) K10 and (**b**) K10-Al-PILC.

The UV–Vis graph (Figure 12) shows the variation in absorbance at 232 nm as a function of adsorbent mass (1–150 mg per 10 mL TNT) for two materials: commercial K10 clay and K10-Al-PILC.

The data point at 0 mg represents the initial TNT solution without any adsorbent, serving as a reference for maximum absorbance. The observed decrease in absorbance with increasing adsorbent mass indicates a progressive reduction in TNT concentration in solution, reflecting enhanced adsorption performance. In the case of K10 (Figure 12a), the absorbance decreases gradually and levels off at relatively high values, even at the maximum tested dose of 150 mg. This trend suggests a limited adsorption capacity, likely attributed to the low specific surface area and the scarcity of active sites on the untreated clay. To account for measurement uncertainty, the original data is accompanied by two symmetrically adjusted series (±0.01), which follow the same downward trend. This consistent behavior across all curves supports the interpretation that adsorption efficiency per unit mass decreases with increasing adsorbent loading, possibly due to surface saturation. Similar limitations have been reported in studies involving montmorillonite used for organic pollutant removal [15].

Figure 12b also shows a clear decreasing trend in absorbance as the adsorbent mass increases, indicating that more TNT is being removed from the solution with higher doses of K10-Al-PILC. The most significant drop in absorbance occurs between 10 mg and 100 mg, after which the curve begins to plateau between 125 mg and 150 mg, suggesting that the adsorption process is approaching saturation. The linear regression line fitted to the upper-bound series demonstrates a strong negative correlation between adsorbent mass and absorbance, with an R^2^ value of 0.9354, meaning that over 92% of the variation in absorbance is explained by the adsorbent mass. The error bars included for each point (±0.02) reflect the experimental uncertainty and provide a visual range of possible variation around each measurement. The consistent downward trend across all three series (original, +0.02, and −0.02) confirms the reliability of the data and the effectiveness of the adsorbent. These results are consistent with previous findings on pillared clays used for nitroaromatic compound adsorption [14,61]. K10-Al-PILC achieves a TNT removal efficiency of 71.98% at 150 mg, significantly outperforming untreated K10 (23.34%), confirming the superior performance of the pillared material under the tested conditions (pH 5.0, 25 °C, 20 min equilibration). This performance aligns with other studies that demonstrate the benefits of pillaring in improving adsorption kinetics and capacity [62].

#### 3.2.2. The Influence of Contact Time

**Figure 13:**
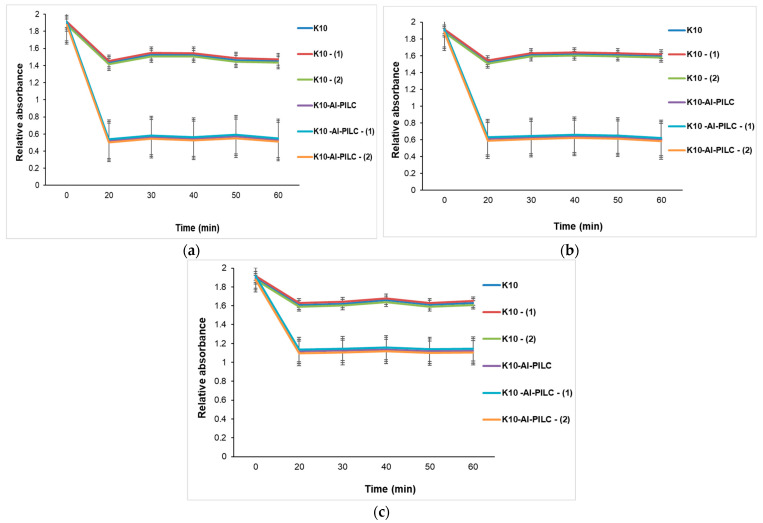
Influence of contact time on TNT absorption at 232 nm from S5 source with different doses of K10 and K10-Al-PILC: (**a**) 50 mg; (**b**) 100 mg; (**c**) 150 mg, used for 10 mL TNT.

At the lowest tested mass (Figure 13a), the difference between the two materials is less pronounced but still evident. K10 shows almost no significant change in absorbance, indicating very limited adsorption capacity. K10-Al-PILC, although less efficient than at higher dosages, still achieves a measurable reduction in absorbance, demonstrating that even small amounts of pillared clay outperform the raw material under identical conditions.

In the case of using 100 mg (Figure 13b), the trends remain consistent. K10 continues to show minimal adsorption, with absorbance values remaining relatively high and stable throughout the contact time. Meanwhile, K10-Al-PILC maintains a significantly better performance, with a clear reduction in absorbance over time. Although the adsorption rate is slightly lower than at 150 mg, the material still demonstrates superior efficiency, confirming that pillaring improves adsorption even at reduced doses.

At the highest tested adsorbent mass (Figure 13c, 150 mg), the difference in adsorption performance between the two materials is most pronounced. The unmodified K10 clay shows a slow and limited decrease in absorbance over time, indicating low TNT removal efficiency. After 60 min, the residual absorbance remains high, suggesting that the material’s compact lamellar structure and low surface area limit the availability of active adsorption sites. In contrast, K10-Al-PILC exhibits a rapid and substantial decrease in absorbance within the first 20 min, followed by a plateau. This behavior reflects efficient and fast TNT adsorption, attributed to the increased porosity and surface area introduced by the aluminum pillaring process, which enhances the accessibility of active sites within the interlayer space.

The three graphs presented in Figure 13 clearly demonstrate that K10-Al-PILC exhibits significantly higher TNT adsorption capacity compared to unmodified K10 clay across all tested adsorbent masses. The enhanced performance is attributed to the structural and textural improvements resulting from the pillaring process, which increases surface area, pore volume, and the density of accessible acid sites. The difference in performance becomes evident within the first 20–30 min of contact and remains consistent throughout the experimental period. These findings support the selection of K10-Al-PILC for further catalytic applications, particularly in ozonation processes where surface accessibility and active site availability are essential.

#### 3.2.3. Influence of pH

**Figure 14:**
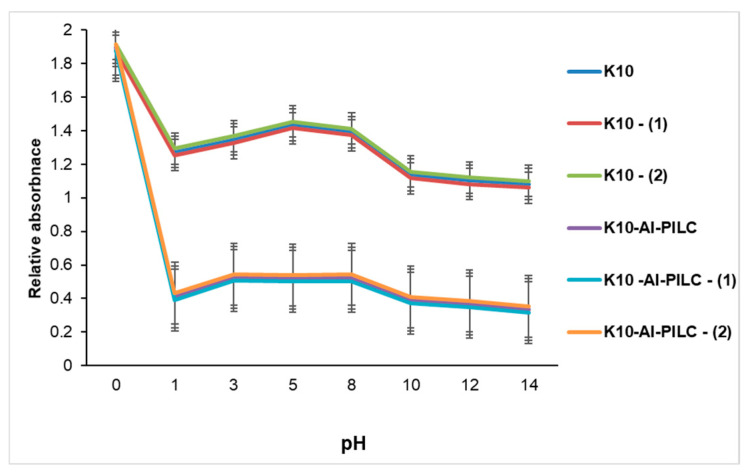
Influence of pH on TNT absorption at 232 nm from S5 source (150 mg) onto K10 and K10-Al-PILC.

The data presented in Figure 14 reveal distinct differences in adsorption efficiency, influenced by both the adsorbent type and the pH of the solution. The commercial K10 material exhibited low adsorption capacity, with minimal changes in absorbance across different pH levels. This limited performance is primarily attributed to its relatively low specific surface area and the scarcity of active sites capable of interacting with TNT molecules.

Under the mildly acidic conditions (pH 5.0–6.5) used in the experiments, TNT predominantly exists in its molecular form. However, the unmodified surface of K10 does not provide sufficient interaction potential to facilitate effective retention of the pollutant. In contrast, the aluminum-pillared material demonstrated significantly enhanced adsorption efficiency, as evidenced by the marked reduction in absorbance values. This improvement is due to structural modifications introduced by the aluminum pillars, which increase both the surface area and porosity of the material. Moreover, the presence of hydroxyl-aluminum species within the interlayer structure promotes polar–polar interactions and hydrogen bonding with TNT molecules, particularly under pH conditions close to those found in natural waters. These findings underscore the essential role of pH in modulating adsorption performance. While pH alone does not substantially affect TNT retention on unmodified K10, its influence is more pronounced and beneficial in the case of the pillared K10-Al-PILC. The superior adsorption characteristics of K10-Al-PILC highlight its potential as an effective material for the remediation of TNT-contaminated water sources. These results are in agreement with previous studies reported in the literature [63,64,65,66], which emphasize the significant role of pH in modulating the adsorption efficiency of pillared clays, particularly aluminum-pillared clays, due to their enhanced surface properties and interaction mechanisms under varying pH conditions. 

### 3.3. Catalytic Ozonation of TNT

To assess the performance of catalytic ozonation in the degradation of TNT, the combined effects of catalyst mass (K10, K10-Al-PILC, and K10-Al-PILC-V), ozone dose (O_3_), and solution pH were systematically investigated, aiming to optimize the reaction conditions for maximum pollutant removal efficiency.

#### Influence of the Catalyst Mass, O_3_ Dose, and Ozonation Time

In this section, the catalytic ozonation performance of three materials: K10, K10-Al-PILC, and the vanadium-doped K10-Al-PILC-V, was evaluated at varying catalyst masses (50, 100, and 150 mg) and ozone doses (0.5, 1.0, 1.5, and 2.0 g O_3_·h^−1^). All experiments were performed in duplicate, and the graph displays both replicate values and their arithmetic means.

**Figure 15:**
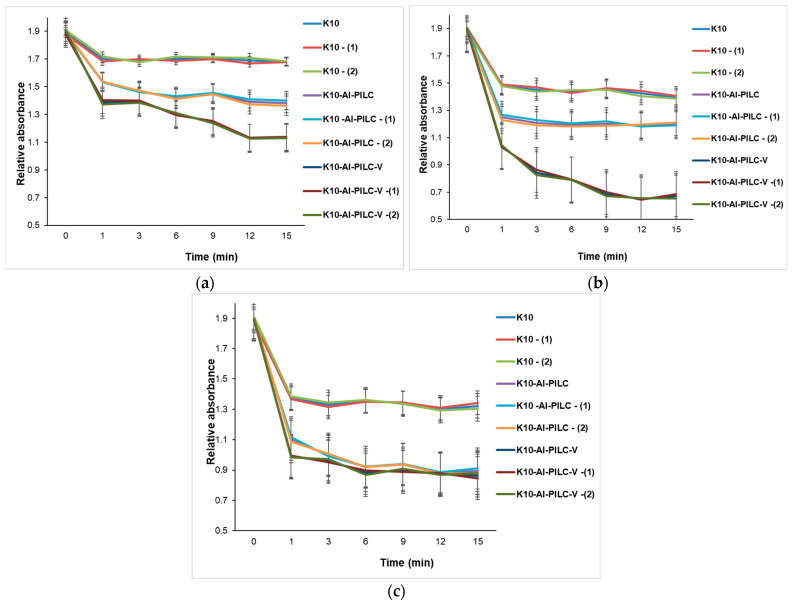
Catalytic degradation of TNT monitored at 232 nm using (**a**) 50 mg; (**b**) 100 mg; and (**c**) 150 mg of K10, K10-Al-PILC, and K10-Al-PILC-V catalysts under 0.5 g O_3_·h^−1^, used for 10 mL TNT.

The progress of the process was monitored by the decrease in absorbance at 232 nm, indicating the degradation of the aromatic compound. At a dose of 50 mg (Figure 15a), the K10 commercial catalyst exhibited low activity, while K10-Al-PILC showed superior efficiency, attributed to the increased surface area and porosity resulting from pillaring. The most effective catalyst was K10-Al-PILC-V, which led to a rapid decrease in absorbance, suggesting efficient generation of reactive oxygen species due to vanadium doping. In Figure 15b, all catalysts showed improved efficiency in the presence of ozone, but K10-Al-PILC-V exhibited the fastest degradation kinetics, confirming the synergy between the pillared structure, vanadium doping, and ozon. Figure 15c showed that the quantity of O_3_ used did not significantly improve the performance of the base catalyst K10 but had a positive effect on the modified catalysts, especially K10-Al-PILC-V, which maintained or even improved its efficiency after treatment.

**Figure 16:**
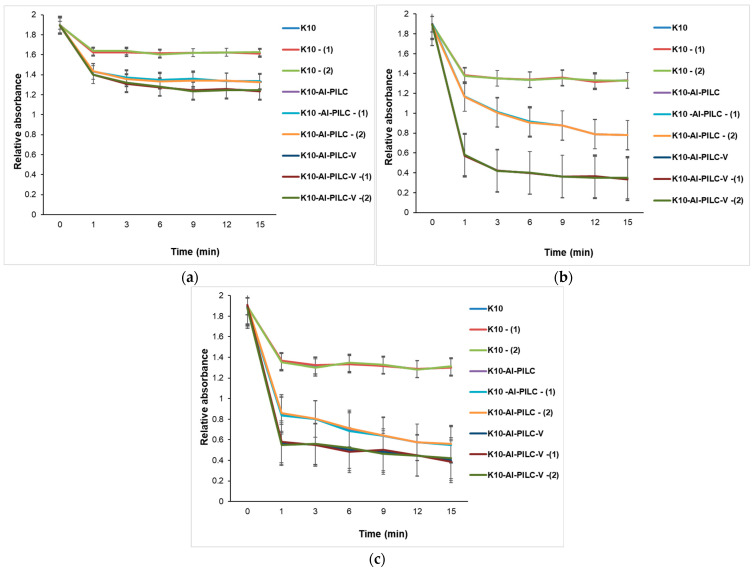
Catalytic degradation of TNT monitored at 232 nm using (**a**) 50 mg; (**b**) 100 mg; and (**c**) 150 mg of K10, K10-Al-PILC, and K10-Al-PILC-V catalysts under 1 g O_3_·h^−1^, used for 10 mL TNT.

Figure 16 illustrates the time-dependent degradation of TNT using increasing amounts of catalyst (50 mg, 100 mg, and 150 mg), highlighting the influence of catalyst type, structural modification, and post-treatment on catalytic performance. Figure 16a shows that the degradation efficiency is modest across all catalysts, with K10-Al-PILC-V showing the best performance. Treatments (T) and (Q) enhance activity, especially for the vanadium-doped catalyst, indicating early-stage benefits of structural and chemical modifications even at low catalyst amounts. Figure 16b shows that increasing the catalyst mass to 100 mg amplifies the differences in performance. K10-Al-PILC-V exhibits the fastest degradation kinetics, confirming the synergistic effect of pillaring, vanadium doping, and surface activation. K10 and its treated forms remain significantly less effective. As shown in Figure 16c, at the highest catalyst loading, K10-Al-PILC-V achieves the most rapid and complete TNT degradation. The performance gap between modified and unmodified catalysts becomes even more pronounced, emphasizing the importance of both catalyst quantity and advanced structural/chemical modifications.

**Figure 17:**
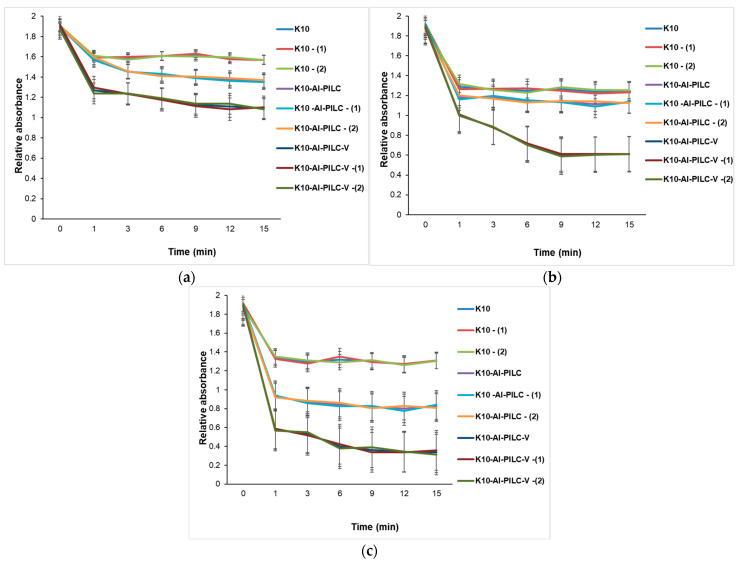
Catalytic degradation of TNT monitored at 232 nm using (**a**) 50 mg; (**b**) 100 mg; and (**c**) 150 mg of K10, K10-Al-PILC, and K10-Al-PILC-V catalysts under 1.5 g O_3_·h^−1^, used for 10 mL TNT.

At 50 mg of the catalyst (Figure 17a), all materials show limited TNT degradation, though vanadium-doped and thermal-treated variants begin to outperform others, indicating early benefits of structural and chemical modifications. At 100 mg (Figure 17b), performance differences become more pronounced. Vanadium-doped catalysts, especially when are thermally treated, exhibit significantly faster degradation kinetics, confirming the synergistic effect of doping, pillaring, and surface activation. Figure 17c shows the case of using 150 mg. This is the most advanced catalyst (K10-Al-PILC-V), achieving over 80% TNT degradation. The performance gap widens, emphasizing that both catalyst quantity and advanced modifications are essential for optimal activity.

**Figure 18:**
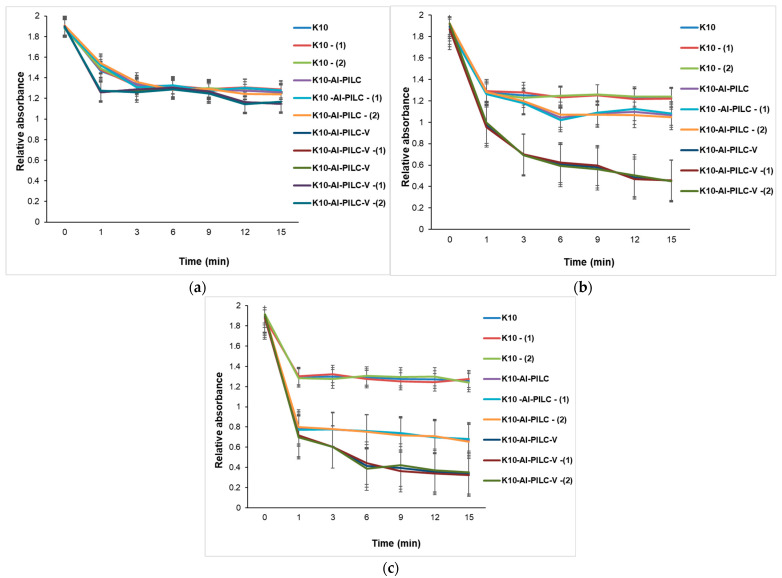
Catalytic degradation of TNT monitored at 232 nm using (**a**) 50 mg; (**b**) 100 mg; and (**c**) 150 mg of K10, K10-Al-PILC, and K10-Al-PILC-V catalysts under 2 g O_3_·h^−1^, used for 10 mL TNT.

The catalytic degradation of TNT, presented in Figure 18, demonstrates a strong dependence on both catalyst formulation and dosage, under a high ozone flow rate of 2 g O_3_·h^−1^. This elevated ozone concentration significantly enhances the oxidative environment, promoting the generation of reactive oxygen species that facilitate TNT breakdown. Unmodified K10 clay exhibits limited catalytic activity, as reflected by the slower decrease in absorbance across all tested masses. In contrast, K10-Al-PILC and especially K10-Al-PILC-V show markedly improved degradation efficiency. These enhancements are attributed to increased surface area, better dispersion of active sites, and the redox properties of vanadium, which likely promote more effective ozone activation. Increasing the catalyst dosage from 50 mg to 150 mg results in faster and more complete TNT degradation, confirming the synergistic effect between catalyst mass and ozone availability. The most efficient system, K10-Al-PILC-V at 150 mg, achieves 82.28% degradation within 15 min, highlighting the importance of both structural optimization and sufficient oxidant supply in advanced oxidation processes.

In Table 4, the TNT removal efficiencies through adsorption and catalytic ozonation are presented for the K10, K10-Al-PILC, and K10-Al-PILC-V materials. The removal efficiencies were calculated using Equation (1), based on the variation in absorbance before and after treatment. Adsorption yields were measured after a maximum contact time of 60 min, while catalytic ozonation efficiencies were determined after 15 min of reaction. The table includes results for three catalyst dosages (50, 100, and 150 mg) and four ozone flow rates (0.5, 1.0, 1.5, and 2.0 g·h^−1^).

## 4. Discussion

The results obtained in this study clearly highlight the significant influence of structural and chemical modifications on the performance of clay-based materials used in adsorption and catalytic ozonation processes. Each material analyzed: K10, K10-Al-PILC, and K10-Al-PILC-V, exhibits distinct characteristics that affect both pollutant retention capacity and the efficiency of reactive species generation required for advanced oxidation.

K10, a commercially available acid-activated montmorillonite clay, was used as a reference material due to its availability. Despite its high specific surface area (220–270 m^2^·g^−^^1^), its performance in both adsorption and catalytic ozonation was modest. The maximum adsorption yield reached only 23.34% at a dose of 150 mg, while the catalytic ozonation efficiency peaked at 35.12% under 2 g·h^−^^1^ ozone. These results suggest that, although K10 possesses a well-developed porous structure, it lacks specific active sites capable of facilitating strong interactions with organic pollutants or catalyzing ozone decomposition. The relatively large average pore diameter (~6 nm) may enhance molecular diffusion but does not necessarily promote effective retention or catalytic activity, limiting the overall process efficiency.

The intercalation of aluminum ions into the clay layers resulted in the formation of a pillared clay (PILC) structure, which stabilized the layered architecture and introduced a more uniform pore network. Although the specific surface area decreased to 186.247 m^2^·g^−^^1^, the pore volume (0.171 cm^3^·g^−^^1^) and reduced average pore diameter (~4.55 nm) indicated a more compact and efficient structure for adsorption. This modification led to a significant increase in adsorption yield, reaching 71.98% at 150 mg. More importantly, the catalytic ozonation efficiency improved substantially, with a maximum of 70.73% at 1 g·h^−^^1^ ozone. These findings suggest that the introduction of active sites via aluminum plays an essential role in ozone activation, facilitating the formation of reactive oxygen species such as hydroxyl radicals. Thus, K10-Al-PILC not only enhances pollutant retention but also accelerates oxidative degradation processes.

Further functionalization of the pillared clay with vanadium resulted in the most catalytically active material in this study. Although the textural parameters (specific surface area of 176.51 m^2^·g^−^^1^ and pore volume of 0.158 cm^3^·g^−^^1^) are similar to those of the aluminum-pillared precursor, the catalytic ozonation yields were significantly higher, reaching a maximum of 82.28%. This improvement is attributed to the presence of redox-active vanadium centers (V^5+^/V^4+^), which are known to efficiently catalyze ozone decomposition into hydroxyl radicals and other reactive oxygen species. The presence of these redox sites enables more efficient ozone utilization, even at moderate doses, potentially reducing operational costs and energy consumption. Additionally, the chemical stability of vanadium within the clay matrix suggests enhanced durability and reusability of the catalyst under repeated uses.

The findings of this study confirm that increasing surface area alone is not sufficient to achieve high performance in advanced water treatment processes. Chemical functionalization of the material surface is essential to introduce active sites, either acidic or redox, that can facilitate both adsorption and catalytic oxidation. In this context, K10-Al-PILC-V emerges as a highly promising catalyst for industrial applications, offering high removal efficiencies, chemical stability, and effective ozone utilization.

Moreover, the marked differences in performance among the three materials underscore the importance of an integrated approach in catalyst design, combining optimized textural properties with tailored surface chemistry. This study thus provides a solid foundation for the development of new hybrid materials tailored for advanced oxidation processes in wastewater treatment, with strong potential for scale-up and real-world implementation.

The results obtained for K10 are consistent with values reported in the literature. The maximum adsorption yield reached 23.34%, while catalytic ozonation achieved up to 35.12%. These values align with those typically reported for natural clays such as kaolinite or bentonite [54], where TNT adsorption rarely exceeds 25% due to low surface area and limited active sites. The modest catalytic performance is also expected, as unmodified clays generally show catalytic ozonation yields below 35% under laboratory conditions [67]. The low efficiency is attributed to the structural limitations of natural clay, which reduce interactions with TNT molecules and reactive ozone species.

The results obtained for K10-Al-PILC confirm the enhanced performance expected from pillared clays. The maximum adsorption yield reached 71.98%, while catalytic ozonation achieved up to 70.73%. These values are consistent with the literature data, where Al-pillared clays typically show TNT removal efficiencies of 60–75%, depending on synthesis conditions and pollutant concentration [68]. The improved performance is attributed to the increased surface area, porosity, and acidity introduced by the pillaring process [69]. These structural enhancements promote stronger interactions with TNT molecules and more efficient catalytic activity during ozonation.

The results obtained for K10-Al-PILC-V confirm the enhanced catalytic performance expected from vanadium-modified pillared clays. Although adsorption yield was not directly measured, catalytic ozonation reached up to 82.28%. This value is consistent with the literature reports, where similar materials have shown degradation efficiencies between 80–90% for various organic pollutants [70,71]. The improved performance is attributed to the synergistic effect of vanadium, which enhances redox activity and promotes the generation of reactive oxygen species during ozonation.

In parallel with the material performance analysis, it is important to consider the environmental implications of TNT contamination in former military or industrial sites. Although TNT can adsorb onto organic matter in the soil, this interaction does not neutralize its toxic potential. The compound remains chemically stable under many environmental conditions and can undergo leaching, microbial transformation, or photodegradation, resulting in the formation of secondary products such as amino-dinitrotoluenes and other nitroaromatic derivatives, which may be equally or more hazardous. These transformation pathways contribute to the long-term persistence of TNT and its by-products in the environment, posing chronic risks to both ecosystems and human health.

In the specific case of the Plaiul Arșiței–Cireșu–Leșunț forest, where the presence of residual explosives has been documented, the area is actively used by local communities and intersected by recreational trails. This context renders passive natural attenuation strategies insufficient. Human intervention through targeted remediation, such as soil excavation, phytoremediation, or advanced oxidation processes, is therefore essential to reduce contaminant levels below regulatory thresholds and ensure public safety.

Furthermore, the potential revitalization of such areas for tourism, housing, or other civilian purposes must be preceded by comprehensive risk assessments and remediation efforts. Only after the complete removal or neutralization of explosive residues can these sites be safely repurposed. In this regard, the development of efficient, low-cost, and environmentally sustainable remediation technologies, such as those based on functionalized clay catalysts, can play a pivotal role in transforming contaminated landscapes into safe and productive spaces.

Ultimately, this study contributes to the broader goal of environmental depollution by proposing effective and sustainable remediation strategies for water contaminated with persistent organic pollutants.

Our findings therefore support active decontamination efforts, not only to eliminate ongoing human exposure risks but also to restore environmental quality.

## 5. Conclusions

This study contributes to the broader goal of environmental depollution, with a particular focus on the remediation of water contaminated by persistent organic pollutants such as TNT. The increasing presence of nitroaromatic compounds in industrial and military wastewater poses serious risks to both human health and ecosystems, highlighting the need for efficient and sustainable treatment technologies.

Through structural and chemical modifications of montmorillonite, we developed advanced adsorbent–catalyst materials with enhanced performance in TNT removal. While unmodified K10 exhibited limited adsorption and catalytic activity, aluminum pillaring introduced active sites and optimized pore structure, improving both properties. Vanadium incorporation further enhanced catalytic ozonation efficiency via redox-active centers, enabling superior ozone activation.

The synergy between surface chemistry and textural properties proved essential for efficient advanced oxidation. K10-Al-PILC-V demonstrated the highest catalytic performance (82.28%) under optimized conditions (1.5 g·h^−1^ O_3_ for 15 min, 150 mg/10 mL of ~10^−4^ M TNT), making it a promising candidate for industrial-scale water treatment technologies. Its high removal efficiency, chemical stability, and reusability support its application in continuous-flow systems for the remediation of nitroaromatic contaminants in wastewater from explosives manufacturing, military facilities, and chemical industries.

Future research should focus on scaling up these materials, optimizing regeneration protocols, and evaluating long-term performance under real operating conditions. Additionally, integrating these materials into modular treatment systems could enhance their applicability in decentralized or mobile water purification units.

## Figures and Tables

**Table 1:** Sampling and physicochemical data of TNT-contaminated water.

Sample ID	Sampling Site	GPS Coordinates	Pretreatment	pH	Water Temperature (°C)	TNT Concentration (M)	Date
S1	Trotuș river, Asău	46.496482 N, 26.373015 E	Freezing −20 °C, 48 h	6.8	8.2	≈10^−5^ M	May 2024
S2	Trotuș river, Dofteana	46.338166 N, 26.535949 E	Freezing −20 °C, 48 h	6.9	9.6	≈10^−4^ M	June 2024
S3	Leșunțul Mare brook, Oituz	46.162721 N, 26.565539 E	Freezing −20 °C, 48 h	6.4	10.1	≈10^−5^ M	July 2024
S4	Sulța brook, Agăș	46.450620 N, 26.205801 E	Freezing −20 °C, 48 h, 30 min	6.8	8.3	≈10^−4^ M	July 2024
S5	Plaiul Arșiței-Cireșu-Leșunț, Oituz	46.166637 N, 26.593792 E	Thermal treatment 70 °C, 30 min	7.4	13.6	≈10^−4^ M	April 2025

**Table 2:** Textural properties of synthesized materials determined by BET analysis.

Sample ID	Specific Surface Area (m^2^·g^−1^)	Total Pore Volume (cm^3^·g^−1^)	Average Pore Diameter (nm)
K10	241.11	~36.0	~6.0
K10-Al-PILC	186.247	0.171	4.549
K10-Al-PILC-V	176.51	0.158	4.564

**Table 3:** The characteristic temperatures and the mass loss of thermal decomposition of the studied materials.

Sample	Stage	T_onset_ (°C)	T_max_ (°C)	T_endset_ (°C)	Δm_i_ (%)	Residue %
K10	I	28.71	35.79	225.80	6.89	89.06
	II	225.8	481.65	528.81	2.29	
	III	528.81	589.28	698.02	1.47	
K10-Al-PILC	I	23.49	38.32	329.83	9.28	87.43
	II	329.83	477.05	521.10	2.24	
	III	521.10	657.12	698.02	1.41	
K10-Al-PILC-V	I	18.75	41.55	331.23	10.55	85.66
	II	331.23	457.69	518.60	2.17	
	III	518.60	670.87	698.02	1.75	

**Table 4:** Summary of TNT removal efficiencies via adsorption and catalytic ozonation, at various catalyst dosages and ozone flow rates.

Adsorbent/Catalyst	Amount (mg)	Adsorption Yield (%)	Ozone Dose (g·h^−1^)	Catalytic Ozonation Yield (%)
K10	50	14.02 ± 0.11	0.5	11.35 ± 0.07
			1	14.52 ± 0.10
			1.5	17.15 ± 0.19
			2	32.64 ± 0.23
	100	15.77 ± 0.13	0.5	26.36 ± 0.173
			1	29.77 ± 0.21
			1.5	34.38 ± 0.24
			2	35.12 ± 0.25
	150	23.34 ± 0.17	0.5	30.23 ± 0.21
			1	31.08 ± 0.22
			1.5	31.08 ± 0.22
			2	33.65 ± 0.23
K10-Al-PILC	50	40.79 ± 0.32	0.5	27.13 ± 0.18
			1	29.75 ± 0.21
			1.5	28.24 ± 0.19
			2	33.63 ± 0.23
	100	68.26 ± 0.52	0.5	36.64 ± 0.26
			1	58.84 ± 0.39
			1.5	40.36 ± 0.28
			2	43.76 ± 0.30
	150	71.98 ± 0.55	0.5	52.84 ± 0.36
			1	70.73 ± 0.47
			1.5	56.48 ± 0.39
			2	64.74 ± 0.44
K10-Al-PILC-V	50	-	0.5	40.21 ± 0.26
			1	34.57 ± 0.24
			1.5	42.37 ± 0.28
			2	38.85 ± 0.26
	100	-	0.5	64.65 ± 0.44
			1	81.95 ± 0.57
			1.5	67.81 ± 0.47
			2	76.02 ± 0.50
	150	-	0.5	54.43 ± 0.37
			1	78.70 ± 0.53
			1.5	82.28 ± 0.56
			2	82.22 ± 0.56

## Data Availability

The original contributions presented in this study are included in the article. Further inquiries can be directed to the corresponding author.
